# Overexpression of microRNA-206 in the skeletal muscle from myotonic dystrophy type 1 patients

**DOI:** 10.1186/1479-5876-8-48

**Published:** 2010-05-20

**Authors:** Stefano Gambardella, Fabrizio Rinaldi, Saverio M Lepore, Antonella Viola, Emanuele Loro, Corrado Angelini, Lodovica Vergani, Giuseppe Novelli, Annalisa Botta

**Affiliations:** 1 Biopathology Department, Tor Vergata University, Rome, Italy; 2 Fondazione Livio Patrizi, Rome, Italy; 3 Pharmacobiological Science Department, "Magna Grecia" University, Catanzaro, Italy; 4 Neurosciences Department, University of Padua, Padua, Italy; 5 Fatebenefratelli Hospital, Villa S. Pietro,. Rome, Italy

## Abstract

**Background:**

MicroRNAs are highly conserved, noncoding RNAs involved in post-transcriptional gene silencing. They have been shown to participate in a wide range of biological processes, including myogenesis and muscle regeneration. The goal of this study is to test the hypothesis that myo-miRs (myo = muscle + miR = miRNA) expression is altered in muscle from patients affected by myotonic dystrophy type 1 (DM1), the most frequently inherited neuromuscular disease in adults. In order to gain better insights about the role of miRNAs in the DM1 pathogenesis, we have also analyzed the muscular expression of miR-103 and miR-107, which have been identified *in silico *as attractive candidates for binding to the *DMPK *mRNA.

**Methods:**

To this aim, we have profiled the expression of miR-133 (miR-133a, miR-133b), miR-1, miR-181 (miR-181a, miR-181b, miR-181c) and miR-206, that are specifically induced during myogenesis in cardiac and skeletal muscle tissues. miR-103 and miR-107, highly expressed in brain, heart and muscle have also been included in this study. QRT-PCR experiments have been performed on RNA from vastus lateralis biopsies of DM1 patients (n = 7) and control subjects (n = 4). Results of miRNAs expression have been confirmed by Northern blot, whereas *in situ *hybridization technique have been performed to localize misexpressed miRNAs on muscle sections from DM1 and control individuals.

**Results:**

Only miR-206 showed an over-expression in 5 of 7 DM1 patients (threshold = 2, fold change between 1.20 and 13.22, average = 5.37) compared to the control group. This result has been further confirmed by Northern blot analysis (3.37-fold overexpression, *R*^2 ^= 0.89). *In situ *hybridization localized miR-206 to nuclear site both in normal and DM1 tissues. Cellular distribution in DM1 tissues includes also the nuclear regions of centralized nuclei, with a strong signal corresponding to nuclear clumps.

**Conclusions:**

This work provides, for the first time, evidences about miRNAs misexpression in DM1 muscle tissues, adding a new element in the pathogenesis of this complex genetic disease.

## Background

Myotonic dystrophy type 1 (DM1; MIM #160900), the most frequent autosomal dominant myopathy in adults, is associated with an expansion of (CTG)n repetitions in the 3'UTR of the *DMPK *gene (DMPK; MIM#605377), on chromosome 19q13.3 [1-3]. Common clinical findings are myotonia, muscle wasting and weakness. Additional features of the disease typically include heart conduction defects, cataracts, hypogonadism, and cognitive impairment [4].

The expanded *DMPK *mRNA play a trans-dominant effect on RNA metabolism through its binding to the Muscleblind-like 1 (MBNL1) splicing regulator, leading to abnormal alternative splicing for a set of genes mainly expressed in skeletal muscle and heart [5,6]. Several expression studies have also been applied to further understand the pathological mechanism occurring in DM1 muscle and they support the idea that the toxic effect of CUG^exp ^RNA may occur also at the level of transcription [7,8]. Less is known about the expression of microRNA genes and DM1. MicroRNAs (miRNAs) are a class of naturally occurring small noncoding RNAs that control gene expression by targeting mRNAs for translational repression or cleavage [9]. Primary miRNA transcripts are cleaved into 70- to 80-nucleotide precursor miRNAs (pre-miRNAs) hairpins by RNase III Drosha in the cell nucleus and transported to the cytoplasm, where pre-miRNAs are processed by RNA Dicer into 19- to 25-nucleotide miRNA duplexes. One strand of each duplex is degraded, and the other strands become mature miRNA, which recognize sites in the 3'-UTR of the target mRNAs and cause translational repression or mRNA cleavage. miRNAs are a new player among gene regulation mechanisms, and their functions have not been fully explored but are known to include the regulation of cellular differentiation, proliferation, and apoptosis [10]. They have been shown to participate in a wide range of biological processes, including myogenesis and muscle regeneration. The three muscle-specific miRNAs, miR-1, miR-133, and miR-206 have been shown to play important roles in the regulation of muscle development [11].

miR-1 and miR-133 are expressed in cardiac and skeletal muscle and are transcriptionally regulated by the myogenic differentiation factors and serum response factor (SRF). The myogenic transcription factors myogenin and myogenic differentiation 1 (MyoD) bind to regions upstream of the miR-1 and miR-133 stem loop, providing a molecular explanation for their observed induction during myogenesis [12-14]. Moreover, miR-1 promotes differentiation of cardiac and skeletal progenitors and their exit from the cell cycle in mammals [15], while miR-133 inhibits their differentiation and maintains them in a proliferative state. miR-206 is expressed only in skeletal muscles, and promotes muscle differentiation if induced by MyoD and myogenin during myogenesis [16,17]. These muscle-specific miRNAs seem to participate in muscle diseases, including cardiac hypertrophy, heart failure, cardiac arrhythmias, congenital heart disease, and muscular dystrophy [18-22]. Other miRNAs, not specifically expressed in muscle, have been proposed to be involved in DM1 pathogenesis. A computational analysis on the repression effects of CTG-repeat binding miRNAs, revealed that miR-103 and 107 are attractive candidates for binding to *DMPK *transcript in a length-dependent manner [23]. In this model, mir-107 and mir-103 which contain CAG repeats in their seed regions, preferentially bind to the mutated *DMPK *mRNA. This could have a miRNA-leaching effect on the amount of unbound miRNA which is reduced and could no longer repress other target genes. miRNAs involvement could therefore have significant consequences on the expression of proteins important in DM1 disease pathogenesis and progression.

The main goal of this study is to test the hypothesis that myo-miRs expression is altered in muscle biopsies from DM1 patients with comparable expansion size. In order to gain better insights about the role of miRNAs in DM1, we have also analyzed the muscular expression of the miR-103 and miR-107 CTG-repeat binding miRNAs.

A combination of Northern blot and QRT-PCR experiments have been utilized to quantify the expression levels of miRNAs, while *in situ *hybridization performed on muscle sections revealed the intracellular localization of misexpressed miRNAs. This is the first report investigating the potential involvement of miRNAs in the pathogenesis of DM1 and shows a significant overexpression of miRNA-206, whose functional significance remains to be elucidated.

## Methods

### Patient recruitment

Seven unrelated DM1-patients, aged 30-50 years, were diagnosed at the Department of Neurology, University of Padua, Padua, Italy. The diagnosis of DM1 was based on clinical, electromyographic (high frequency repetitive discharges), ophthalmologic and cardiac investigations. After written informed consent, DM1 muscle samples were obtained by diagnostic needle biopsies from vastus lateralis. Control samples (vastus lateralis) were obtained from 4 subjects deemed free of neuromuscular disorders, aged 35 and 42 years. All muscle biopsies were frozen in liquid nitrogen immediately after surgery, and stored at -80°C until used. Histological analysis of DM1 biopsies showed the typical pathology of the disease, including atrophic fibres with increased fibre size variation and marked proliferation of centrally located nuclei. Hematoxylin-eosin and Ghomory thricome stains showed absence of inflammatory aspects in all the DM1 samples analyzed. The main pathohistological features of each DM1 specimens used in this study are reported in Table 1. (CTG) repeat expansion sizes were determined in muscle tissues and resulted to be included into the E2 class. The study was approved by the local ethical committee of Tor Vergata University and all the procedures have been performed in compliance with the Helsinki Declaration.

**Table 1:** Pathohistological features of each DM1 specimens

Patients	Sex	Muscle	Lysosomal activity*	Muscle pathohistological aspects
DM1-1	M	VL	xxxx	atrophy
DM1-2	M	VL	xxx	atrophy
DM1-3	M	VL	xx	mild atrophy
DM1-4	M	VL	xxxx	atrophy
DM1-5	M	VL	x	few atrophic fibers
DM1-6	F	VL	xxxx	severe atrophy
DM1-7	M	VL	absent	few atrophic fibers

Are shown the main pathohistological features of each DM1 specimens used in this study (sex, muscle, lysosomal activity and muscle pathohistological aspects). Muscle biopsies from VL of 1 female and 3 males have been used as control samples. (VL = Vastus Lateralis; M = Male; F = Female; * = Phosphatase activity present in atrophic fiber where autophagy is active.)

### Quantitative reverse transcription-PCR of miRNAs and mRNAs

Total RNA was extracted from 500 mg of frozen vastus lateralis tissue using TRIZOL reagent (Life Technologies, Inc.) following the manufacturer's instructions. cDNA was reverse transcribed from 3 μg of total RNA samples using specific miRNA primers and reagents from the TaqMan MicroRNA Reverse Transcription kit and Assays (Applied Biosystems). The resulting cDNA was amplified by PCR using TaqMan MicroRNA Assay primers with the Taq Man Universal PCR Master Mix (code 4324018) and analyzed with a 7500 ABI PRISM Sequence Detector System according to the manufacturer's instructions (Applied Biosystems). We analyzed the expression of the following miRNAs: hsa- mir-1 (Assay n. 4373161), hsa- mir-206 (Assay n. 4373092), hsa- mir-181a (Assay n. 4373117), hsa- mir-181b (Assay n. 4373116), hsa- mir-181c (Assay n. 4373115), hsa- mir-133a (Assay n. 4373142), hsa- mir-133b (Assay n. 4373172), hsa- mir-103 (Assay n. 4373158), hsa- mir-107 (Assay n. 4373154). 7 DM1 patients and 4 control subjects have been included in this study. Values of DM1 patients were compared to the medium value of control subjects analyzed separately. The relative levels of miRNA expression were calculated and normalized using the 2^-ΔΔCt ^method relative to HSA-let-7a miRNA (Assay n. 4373169). All TaqMan-PCRs were performed in triplicates. Both let-7a and U6-sn-RNA were considered initially as possible control miRNAs for normalization of samples. Let-7a miRNA is frequently used as internal control because of its stable expression across human tissues and cell lines [24,25], even though some studies report its misregulation especially in cancer conditions (lung cancer, chronic lymphocytic leukemia, breast cancer, prostate cancer, hepatocellular carcinoma) not related with muscular disorders [26-28]. QRT-PCR analysis (data not shown) showed a similar expression level of U6 and let-7a miRNAs in all the samples included in the study. Finally, let-7a has been chosen as control miRNA because its Ct (Ct = cycle threshold, defined as the number of cycles required for the fluorescent signal to cross the threshold) value is more comparable to the Ct values of myo-miRs considered in this study. For quantification of *Utrophin *transcritpt, 2 μg of total RNA was reverse transcribed using high capacity cDNA reverse transcription kit (Applied Biosystem). The resulting cDNA was amplified using the ABI Prism 7000 Real-Time Sequence Detection System quantification employing the Syber-Green assay. Primers sequence for *Utrophin *was taken from Arning *et al*. [29] (Forward 5' aaggacctggtcaacgttcca 3', Reverse 5' acccgtgtcatagacattgagca 3'). The *Beta-Actin *mRNA level was used as control for normalization of samples (Forward 5#8242; gacaggatgcagaaggagattact 3', Reverse 5#8242; tgatccacatctgctggaaggt 3').

### Nothern Blot analysis

Given the limited amount of RNA available, total RNAs from DM1 muscle biopsies were pooled into two groups: DMa (DM1-1, DM1-2, DM1-3, DM1-4) and DMb (DM1-5, DM1-6, DM1-7). RNAs from 4 healthy subjects were pooled and used as control. RNA was separated on denaturating polyacrylamide gels in TBE buffer, transferred to a nylon membrane (Hybond-N+, GE Biosciences) with Trans-blot SD Semi-dry Transfer Cell (Bio-Rad) and fixed in the membrane by UV crosslinking, with 1200 μJ. Hybridization probes were prepared with 20 μM oligonuclotides, whose sequences were complementary to investigated miRNAs. Probes were labeled with [32P] γ-ATP (5000 ci/mmol; 10 mCi/ml, from Hartmann Analytic GmBH, Germany) using polynucleotide kinase (New England Biolabs). The labeled probes were purified with Sephadex G25 spin columns (GE Biosciences). After adding the probe, hybridization was carried out overnight at 42°C. After hybridization, membranes were washed with SSPE 6×. Dried membranes were exposed to Phosphoimaging plates (Kodak), which were read out in a Storm scanner (Amersham- GE Biosciences). For Northern blot analysis miRNA U6 were used as control for normalization of samples. U6, widely used in Northern Blot analysis, has been chosen as control miRNA because its band intensity is more comparable to the those of myo-miRs considered in this study. Densitometry of autoradiograms was performed using OptiQuant image analysis software (Packard). A linear regression has been applied in order to correlate expression values obtained with QRT-PCR and Northern Blot analysis.

### Western blotting

Muscle 20 μm sections were collected from frozen bioptic samples, lysed in Laemmli buffer and run in a 4-12% T30C4 SDS-PAGE. Proteins were then blotted into nitrocellulose membrane, probed with specific Utrophin (Novacastra, NCL-DRP2) and α-Tubulin (Santa Cruz, B7 sc-5286) antibodies. After incubation with secondary HRP-conjugated antibodies, recognized bands were visualized by chemiluminescence (GE HealthCare). Integrated optical density of each band was calculated with commercial software and normalized compared to Tubulin amounts.

### *In situ *hybridisation

*In situ *hybridization was performed on transversal sections of vastus lateralis muscles from DM1 patients showing a significant up-regulation of miR-206 and from two control subjects included in this study. A locked nucleic acid (LNA) detection probe for miR-206 (Exiqon Cod. EX100008999901), a LNA U6 positive control probe (Exiqon Cod. EX9900201) and a LNA negative control probe with a scramble sequence (Exiqon Cod. EX9900401) have been used in this analysis. All probes were labeled with digoxigenin (DIG) (Roche). Cryosection prepared from quadriceps vastus lateralis of human biopsies in normal and DM1 patients fixed with 4% PFA, were treated with proteinase K, re-fixed with PFA and then acetylated with acetylation buffer (0.1 M triethanolamine pH 8.0).

After washing with PBS and pre-hybridization, slides were incubated with DIG-labeled LNA miR-206 probe, DIG-labeled LNA U6 probe (positive contol) and a DIG-labeled LNA scrambled sequence (negative control) at 49°C overnight. Washes were done at 49°C in 5× SSC, 50% formamide, 2× SSC and at room temperature in 0.2× SSC and then PBS 1×/0.1% Tween-20, then slides were incubated with blocking solution (PBS 1×/0.5% BSA/1 - 5% inactivated FCS), followed incubation with FITC-coupled anti-digoxygenin antibody (Roche) at 4°C overnight.

After washes with PBS 1×/0.1% Tween 20, slides were rinsed with DAPI, mounted and analyzed by fluorescent microscopy Olympus BX51 at 40× magnification.

## Results

### miR-206 expression is increased in DM1 muscle

In this work we have profiled the expression of miR-133 (miR-133a, miR-133b), miR-1, miR-181 (miR-181a, miR-181b, miR-181c) and miR-206, specifically induced during myogenesis, in muscle biopsies from 7 DM1 patients, compared with 4 control subjects. In order to gain better insights about the role of miRNAs in the DM1 pathogenesis, we have also analyzed the muscular expression of miR-103 and miR-107, which have been identified *in silico *as attractive candidates for binding to the *DMPK *target mRNA.

**Figure 1:**
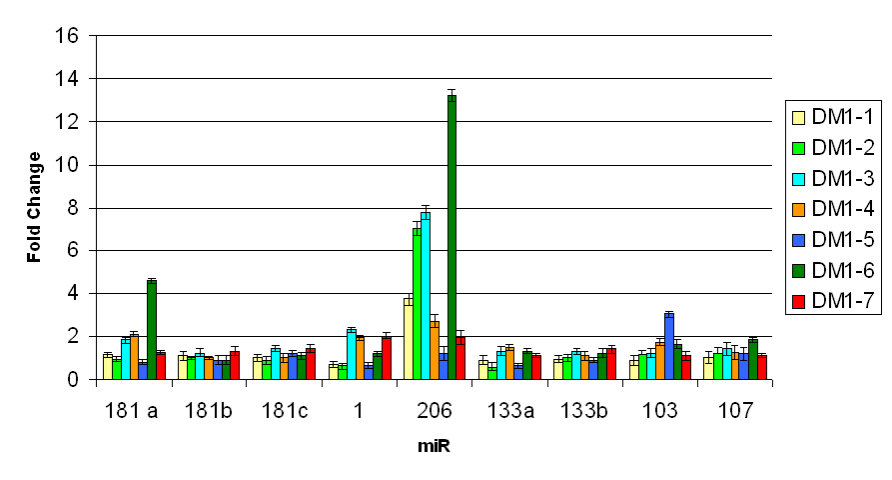
**QRT-PCR quantification of myo-miRs and miR-133 and miR-107 in biopsies from vastus lateralis of 7 DM1 patients compared with 4 controls**. Fold change values of miR-206: DM1-1 = 3,73; DM1-2 = 7,02; DM1-3 = 7,77; DM1-4 = 2,70; DM1-5 = 1,20; DM1-6 = 13,22; DM1-7 = 1,95

**Figure 2:**
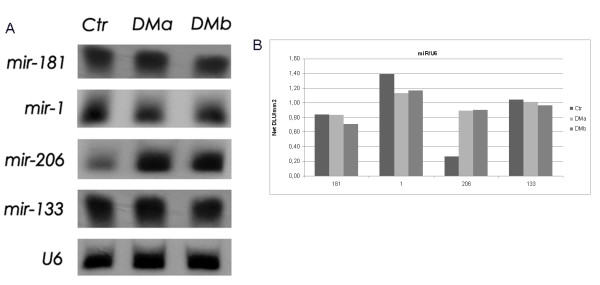
**Northern blot analysis of myo-miRs expression**. DM1 patients were pooled into two groups: DMa (DM1-1, DM1-2, DM1-3, DM1-4) and DMb (DM1-5, DM1-6, DM1-7), 4 healthly subjects were pooled as well. The U6-snRNA was used as control for normalization of samples. Figure 2a: Northern Blot results of the 3 pooled samples (Ctr, DMa and DMb) for the 4 miRNA analyzed (miR 181, miR 1, miR 206 and miR 133) compared to U6-snRNA. Figure 2b: Densitometry of autoradiograms performed using OptiQuant image analysis software (Packard) showing miR/U6 ratios.

We first calculated the relative amount of miRNAs expression using the HSA-let-7a miRNA for normalization of samples in three independent QRT-PCR reactions. As shown in Figure 1, QRT-PCR experiments showed no differences in the expression of miR-1, miR-133, miR-181, miR-103 and miR-107 between DM1 and control muscles. In striking contrast, miR-206 expression was increased in 5 of 7 DM1 patients (threshold = 2, fold change between 1.20 and 13.22, average = 5.37) compared to median value of controls group set as 1 (Fold change of Ctr-2, Ctr-3 and Ctr-4 normalized with Ctr-1 are 1.3, 0.98 and 1,15 respectively). To validate the over-expression of miR-206 in DM1 muscles, we performed Northern blot analysis using pooled DM1 and control samples and U6 as control sn-RNA. We decided to pool samples because the quantity of RNA derived from patients' biopsies was not enough to analyze each sample separately. Figure 2a shows Northern blot results of the four myo-miRs considered (miR 181, miR 1, miR 206 and miR 133) compared to U6-snRNA in DM1 and controls muscle samples. Densitometry analysis of autoradiograms (Figure 2b) further confirmed the results obtained through QRT-PCR. miR-206 was over-expressed in both DMa and DMb pool (DMa = 3,36 +/- 0.11, DMb = 3,39 +/- 0.10). Linear regression demonstrates a statistically significant positive correlation between QRT-PCR and Nothern blot analyses results (R^2 ^DMa = 0.98; R^2 ^DMb = 0.82).

### mRNA and protein level of Utrophin are not decreased in DM1 muscle lysates

**Figure 3:**
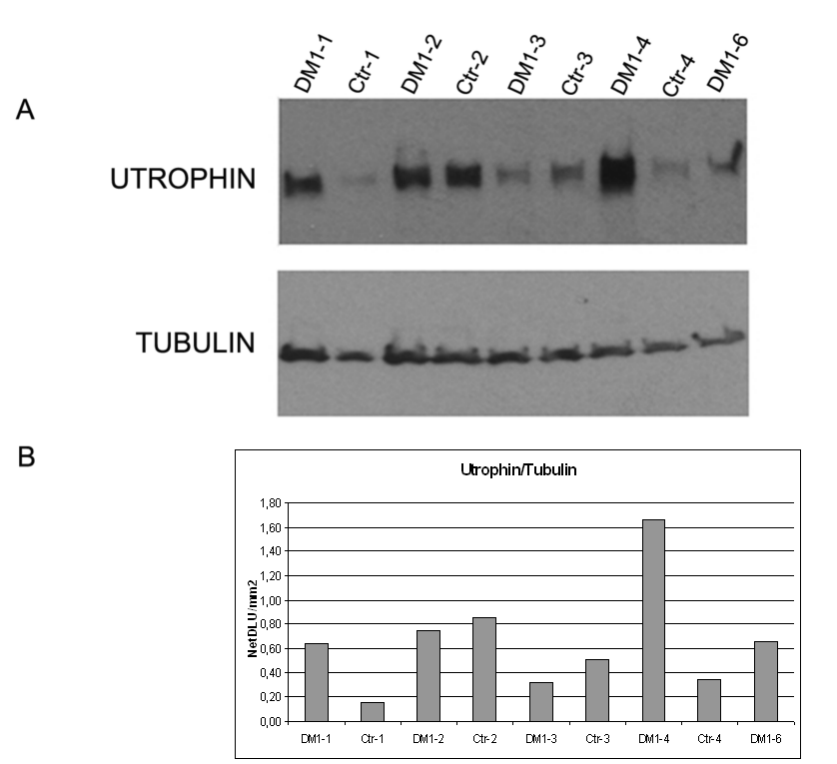
**Western blot analysis showing the Utrophin and α-Tubulin protein expression levels in 5 DM1 patients and 4 controls**. Utophin/Tubulin ratios in the analyzed samples are: DM1-1 = 0,63, DM1-2 = 0,74, DM1-3 = 0,31, DM1-4 = 1,66, DM1-5 = 0,66, CTR 1 = 0,15, CTR 2 = 0,85, CTR 3 = 0,50, CTR 4 = 0,34. Figure 3a: Western blot of Utrophin and α-Tubulin protein expression in the 5 DM1 patients showing the miR-206 upregulation and in 4 controls. 50 μg of sample was loaded on each lane. Figure 3b: Densitometric analysis of Western blot autoradiograms performed using OptiQuant image analysis software (Packard) showing Utophin/Tubulin ratios.

A predicted target gene of miR-206 is the *Utrophin *gene (Utrn) http://microRNA.sanger.ac.uk. Rosenberg et al. [16] confirmed this prediction with multiple lines of evidence indicating that miR-206 acts at post-trascriptional level in repressing *Utophin *expression. We therefore performed Western blot analysis to test the expression levels of the Utrn protein in DM1 patients *vs*. controls. Figure 3a shows a Western blot image for the quantification of Utrophin and Tubulin (used as housekeeping protein) in 5 DM1 patients and 4 controls. We included in this experiment only DM1 patients showing a significant over-expression of miR-206 (DM1-1 fold change 3,73, DM1-2 fold change 7,02, DM1-3 fold change 7,77, DM1-4 fold change 2,70, DM1-6 fold change 13,22). After densitometric analysis of each band, we found a high variability in the Utrophin level both in controls and DM1 patients (Figure 3a). Utrophin/Tubulin ratios range from 0,31 and 1,66 (medium value = 0,80) in DM1 muscles and from 0,15 to 0,85 (medium value = 0,46) in control samples, with no significant differences between the two groups (Figure 3b). A linear regression analysis comparing Utrophin quantification and miR-206 expression did not show any correlation (R^2 ^= 0.132, not shown) indicating that, in our DM1 samples, the levels of Utrophin are not under the direct control of miR-206 expression.

Although miRNAs are believed to regulate their targets primarily through translational inhibition, there is increasing evidence that miRNAs can also influence the abundance of target mRNAs [30]. On this basis, we have also studied the abundance of *Utrophin *mRNA in our muscle specimens. QRT-PCR experiments, using Syber-Green assay and the *Beta-Actin *mRNA as control for normalization of samples, showed no significant differences between DM1 and controls groups (Fold change DM1-1 = 1,27, DM1-2 = -1,82, DM1-3 = 1,77, DM1-4 = 2,1, DM1-5 = 1,06, DM1-6 = -1,92, DM1-7 = 1,29). Again, linear regression analysis comparing *Utrophin *mRNA quantification and miR-206 expression did not show any correlation (R^2 ^= 0.138).

### miRNA-206 localizes to centralized nuclei and nuclear clumps in DM1 muscle sections

**Figure 4:**
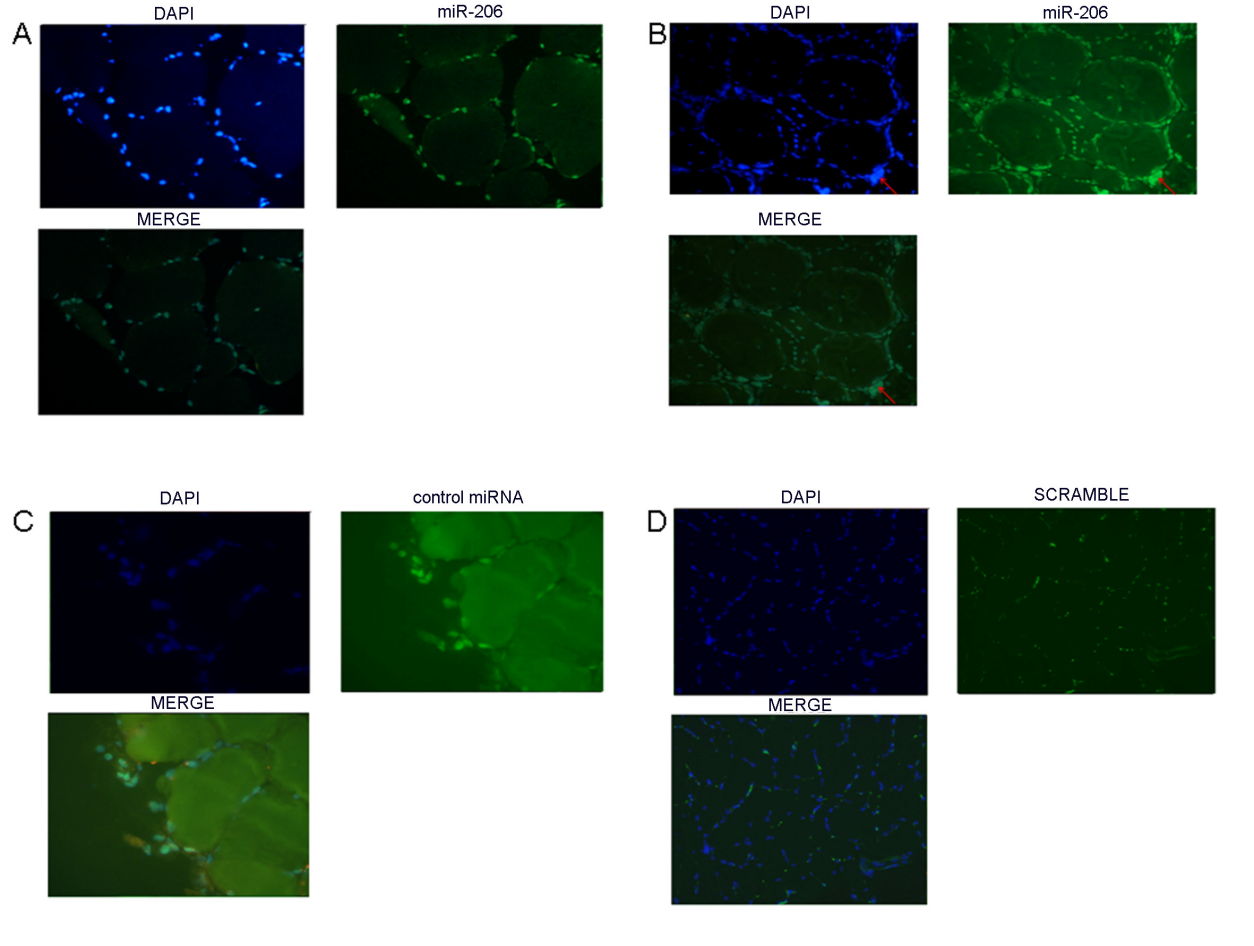
***In situ *hybridisation showing miR-206 localization in transversal section of vastus lateralis muscle from one DM1 patient and one control subject**. 4a: Tissue distribution of miR-206 in an healthy subject. miR-206 was expressed mostly in nuclear regions. 4b: Tissue distribuition of miR-206 in a DM1 patient. The miR-206 strongest signal corresponds to nuclear clumps (red arrow). Expression of miR-206 was also observed in nuclear regions of centralized nuclei. 4c: hybridization of U6-siRNA LNA used as positive control. 4d: LNA probe with a scrambled sequence, which is not present in the human genome, has been used to test the specificity of the probes. Green signal corresponds to lipofuscin-derived autofluorescence of the muscle tissue and does not localize with the nuclei.

To detect the intracellular localization of miR-206, we performed *in situ *hybridization using locked nucleic acid (LNA) probes on cryostat vastus lateralis muscle sections from controls and DM1 patients. Figure 4 shows the hybridization pattern of miR-206 in transversal muscle sections from a DM1 (Figure 4b) and a control (Figure 4a) subject using a DIG-labeled LNA probe detected with a FITC coupled anti-digoxygenin antibody. miR-206 localizes most exclusively to the nuclear region both in normal and DM1 tissues. However, in DM1 muscles a strong signal was detected also in correspondence to centralized nuclei and nuclear clumps (Figure 4b, see red arrow), which are pathological hallmarks of dystrophic muscles. We also investigated expression of miR-206 in cytoplasm both in normal and DM1 tissue, but no signals were visible, indicating a nuclear specific function of miR-206 in the muscle tissue. As controls of hybridization, the muscle sections were hybridized with the LNA U6 positive control probe (Figure 4c), which recognize a small and stable ribonucleoprotein in all human cells. The specificity of hybridization was assessed using an LNA probe with a scrambled sequence not present in the human genome (Figure 4d).

## Discussion

miR-206 is a member of the muscle-specific miR-1 family, that consists of six members clustered into three bicistronic pairs arising from an initial local gene duplication which produced the original paralogous gene cluster (miR-1 and miR-133). Then two "non-local" genomic duplications resulted in the new clusters located on different chromosomes [31]. It is the unique myomiR exclusively expressed in skeletal muscle [32-36] and has been rarely detectable in the heart [37-41]. The skeletal muscle-specific expression of miR-206 was first clearly demonstrated by microarray analysis and later confirmed by Northern blot [16].

Additional muscle-enriched miRNAs have also been identified and shown to be involved in cardiogenesis, myogenic, differentiation and growth [18]. Several studies were performed to analyze the expression of miRNAs, in general, and myo-miRs, specifically, in muscolar dystrophies. Eisenberg *et al*. [22] performed a microarray analysis on 10 muscular disorders in humans, not including DM1. They identified 185 miRNAs with differential expression, but myomiRs were not included in this list. Microarray analyses of muscle from the dystrophin-deficient (*mdx*) mouse, an animal model of Duchenne muscular dystrophy (DMD), suggest that changes in miRNAs expression may contribute to the pathophysiology of muscular dystrophy [42-45]. Therefore, McCarthy *et al*. [46] analyzed the expression of the muscle-enriched miRNAs in the *mdx *diaphragm, the most severely affected muscle in the dystrophin-deficient mouse. They observed an increase in miR-206 expression in this muscle, associated with a similar increase in *Myod1 *expression. These results suggested that miR-206 expression contributes to the chronic pathology observed in the *mdx *diaphragm by repressing expression of genes that otherwise would serve a compensatory function, limiting the severity of the disease, as in the hindlimb musculature [46].

The main goal of this paper was to investigate the pathophysiological roles of muscle-specific miRNAs in DM1, the most frequent autosomal dominant myopathy in adults. We therefore profiled the expression of miR-133, miR-1, miR-181 and miR-206, in 7 vastus lateralis biopsies from DM1 patients compared with 4 control subjects. We have also included in our study the muscular expression of the miR-103 and miR-107 CTG-repeat binding miRNAs which are highly expressed in brain, heart and muscle [23]. These two miRNAs contain CAG repeats in their seed regions and have been identified, through computational analysis, as potential repressor factors of the wild type and mutant *DMPK *transcripts. The binding of miR-103 and miR-107 to the 3'UTR of the *DMPK *expanded mRNAs could therefore affects the stoichiometry of free to bound CTG-repeat binding miRNAs, or otherwise disrupt the CTG-repeat binding miRNA function in DM1 muscle tissues.

After a combination of QRT-PCR and Northern blot experiments, only miR-206 was found to be over-expresssed in 5 of 7 DM1 patients compared with the controls group. Interestingly, samples DM1-5 and DM1-7, which did not show upregulation of miR-206, demonstrated lower phosphatase activity and milder atrophy compared to the other DM1 specimens. The misregulation of miR-206 in DM1 is consistent with what observed by McCarthy *et al*. [46] in the affected diaphragm of *mdx *mouse. Since the vastus lateralis from DM1 patients exhibits all the pathological hallmarks of a dystrophic tissue, miR-206 may contribute to the chronic course of both muscular dystrophies. Several computational and functional studies identified the putative targets of miR-206. Rosenberg *et al*. [17] have predicted its targets based on sequence match, and indicated the p180 subunit of DNA polymerase α and three other genes as direct targets. Down-regulation of the polymerase inhibits DNA synthesis, an important component of the differentiation program, connecting miR-206 function to the cell quiescence in the differentiation process. Moreover they showed that miR-206 was capable of post-transcriptionally repressing Utrophin expression. They concluded that these data could be used to develop specific therapies aimed at increasing or maintaining Utrn expression in Duchenne muscular dystrophy.

To determine whether miR-206 might function in a similar fashion under dystrophic conditions, John J. McCarthy et al. measured *Utrophin *protein levels in *mdx *diaphragm [46]. In this study the *Utrophin *transcript level has also been evaluated, since there is increasing evidence that miRNAs can also accelerate target mRNA degradation [30] with the consequent decreasing of target mRNA abundance. Results indicate that *Utrophin *is post-transcriptionally regulated in the *mdx *diaphragm, but are not consistent with regulation by miR-206 as Utrophin protein increased, not decreased as would be expected if regulated by miR-206. Similarly, we tested the protein and mRNA levels of *Utrophin *in muscle biopsies from DM1 patients showing a miR-206 over-expression. Western blot and QRT-PCR analyses did not demonstrate significant differences between DM1 and controls groups. Our observation further support the idea that the *Utrophin *gene is not target of miR-*206 in vivo *in our DM1 muscle samples.

Hypothetical mRNA targets of miR-206 can also be derived, trough computational analysis, from microarray studies of mRNA differentially expressed in DM1 tissues. Osborne *at al*. [8] performed a global mRNA profiling in transgenic mice that express CUGexp RNA to identify DM1-affected genes and study mechanisms for dysregulation. 175 transcripts were dysregulated in this mice models, comprising 110 transcripts that were upregulated and 65 that were downregulated. *In-silico *analysis through Targetscan http://www.targetscan.org indicate that five of the downregulated transcripts are potential target of miR-206: *RETSAT *(all trans retinol 13,14 reductase), *GNPNAT1 *(glucosamine-phosphate N-acetyltransferase 1), *LAPTM4B *(lysosomal-associated protein transmembrane 4B), *IGFBP5 *(insulin-like growth factor binding protein 5) and *VASP *(vasodilator-stimulated phosphoprotein) mRNAs. It is therefore possible that the effect of miR-206 upregulation found in our DM1 sample could influence the expression of additional genes not reported so far in literature.

Even if data about the target of miRNAs are increasing, less is know about the distribution and localization of miRNAs in the cells. Politz *et al*. described the intracellular localization of miR-206 in single cultured myogenic cells using *in situ *hybridization followed by high-resolution imaging microscopy. They found that miR-206 is not only distributed throughout the cytoplasm as expected but also is concentrated in the nucleolus [47].

To detect and localize miR-206 in our DM1 and control muscle biopsies, we exploited the higher specificity and hybridization efficiency of locked nucleic acid (LNA) probes. These LNA-modified molecules exhibit unprecedented thermal stability when hybridized with their RNA target molecules. The analysis of miRNAs accumulation in frozen tissue sections using (DIG)-labeled LNA probes resulted in the generation of comprehensive miRNA expression atlases that have proven highly useful for functional studies of individual miRNA [48].

We therefore utilized the same technology to determine the tissue localization of miR-206 in transversal section of vastus lateralis from DM1 and control subjects. Interestingly, we found that miR-206 is prevalently expressed in the nuclear regions, with a tissue distribution in DM1 muscles characterized by a strong signal corresponding also to the nuclear clumps and centralized nuclei. The localization of miR-206 in DM1 atrophic fibers may indicate a possible involvement of miR-206 in the process of atrophy which already involves the activation of the MyomiRs network in the regulation of slow myosin expression [46].

Also if deeper studies need to be performed in order to improve our knowledge on miR-206 involvement in DM1, it is possible to speculate that miR-206 could contribute to the chronic course of the pathology and need to be considered for future molecular therapies.

## Abbreviations

QRT-PCR: Quantitative Real Time-Polymerase Chain Reaction; DM1: Myotonic Dystrophy Type 1; UTR: Untranslated Region; DMPK: Dystrophia Myotonica Protein Kinase; MBNL1: Muscleblind-like 1; EXP: Expansion; SRF: Serum response factor; UTRN: Utrophin

## Competing interests

The authors declare that they have no competing interests.

## Authors' contributions

SG: conceived the study design, handled biological samples, performed qrtPCR, analysis and drafted the manuscript, FR participated in the design of the study and performed Northern Blot analysis, SML performed in-situ hybridisation, AV participated in the design of the study and collected the clinical data of patients, EL performed western blot analysis, CA and LV performed clinical analysis and sample collection, GN and AB coordinated the study and participated in manuscript writing and editing. All authors read and approved the final manuscript.
